# Management strategy for children with ovarian immature teratoma: results from a tertiary pediatric oncology center

**DOI:** 10.1186/s12957-024-03452-z

**Published:** 2024-07-04

**Authors:** Gehad Ahmed, Sahar Ahmed Khalil, Maged Elshafiey, Nihal Abdelfattah, Mohamed Eid, Al-Shaimaa Zakaria, Madeeha Elwakeel, Ahmed Elgendy

**Affiliations:** 1 General Surgery Department, Faculty of Medicine, Helwan University, Cairo, Egypt; 2 Surgical Oncology Department, Children’s Cancer Hospital 57357, Cairo, Egypt; 3 Pediatric Oncology Department, National Cancer Institute - Cairo University, Cairo, Egypt; 4 Pediatric Oncology Department, Children’s Cancer Hospital 57357, Cairo, Egypt; 5 Surgical Oncology Department, National Cancer Institute - Cairo University, Cairo, Egypt; 6 Department of Research, Children’s Cancer Hospital 57357, Cairo, Egypt; 7 Pathology Department, National Cancer Institute - Cairo University, Cairo, Egypt; 8 Pathology Department, Children’s Cancer Hospital 57357, Cairo, Egypt; 9 Radio-Diagnosis Department, National Cancer Institute - Cairo University, Cairo, Egypt; 10 Radio-Diagnosis Department, Children’s Cancer Hospital 57357, Cairo, Egypt; 11 Surgical Oncology Unit, Department of Surgery, Faculty of Medicine, Tanta University, 35 Ali Beek Elkbeer street, Tanta, 31515 Egypt

**Keywords:** Ovary, Immature teratoma, Surgery, Adjuvant chemotherapy, Relapse

## Abstract

**Objectives:**

We present an Egyptian study on pediatric ovarian immature teratomas (ITs), aiming to clarify our treatment strategy selection.

**Methods:**

A retrospective review of all children with pure ovarian ITs who were treated at our institution between 2008 and 2023. The analysis included clinical characteristics, tumor staging according to Children’s Oncology Group (COG), grading based on the Norris system, management, and outcomes.

**Results:**

Thirty-two patients were included, with a median age of 9 years. All patients underwent primary surgery. Unilateral salpingo-oophorectomy was performed in 31 patients. Surgical staging was completed in all patients. Based on COG staging, there were 28 patients (87.5%) stage I, 1 (3%) stage II, and 3 (9.5%) stage III. According to Norris classification, 16 patients (50%) were classified as grade I, 9 (28%) grade II, and 7 (22%) grade III. All patients in stage I were treated using surgery-alone approach, whereas the remaining four (12.5%) received adjuvant chemotherapy. Five patients in stage I had gliomatosis peritonei (GP), and none of them underwent extensive surgery. At a median follow-up of 86 months, two patients had events. The first patient (stage III/grade I) developed IT relapse on the operative bed, and the second (stage I/grade I) had a metachronous IT on the contralateral ovary. Both patients were successfully managed with surgery followed by second-line chemotherapy. Five-year overall survival and event-free survival for all patients were 100% and 93.4%, respectively.

**Conclusions:**

Surgery-alone strategy with close follow-up achieves excellent outcomes for localized ovarian ITs in children, irrespective of the Norris grading or the presence of GP. However, adjuvant chemotherapy is questionable for patients with incompletely resected or locally advanced tumors, and its role requires further evaluation through prospective multicentric studies with a larger sample size.

## Introduction

Ovarian tumors in childhood are uncommon entities, and approximately 60–80% of them are of germ cell origin [1–3]. Among such tumors, mature teratomas are the most common, whereas immature teratomas (ITs) are infrequent [4, 5]. Ovarian ITs have a distinct biological structure as they contain variable amounts of neuroepithelial tissue. They are graded based on the proportion of immature tissue components according to the Norris histological grading system [6]. In some patients, ITs may be associated with gliomatosis peritonei (GP), which is the deposition of mature glial tissue on the peritoneal surface [7]. Initially, the pathogenesis of GP was thought to be related to tumor rupture, but subsequent research revealed that GP is mostly caused by reactive metaplasia of submesothelial cells and is genetically unrelated to the primary ovarian tumor [7, 8].

Surgery is the principal treatment modality for ovarian ITs, and complete tumor resection is crucial [9]. There are significant differences in the use of adjuvant chemotherapy between adult and pediatric oncologists [7, 9–11]. In adults, such tumors are considered malignant germ cell tumors, and post-operative platinum-based chemotherapy is routinely administered for all patients except stage IA, grade 1 tumors, based on the International Federation of Gynecology and Obstetrics staging system (FIGO) [12, 13]. However, in children, there is no consensus regarding the indications of adjuvant chemotherapy, and its role remains debatable [5]. Factors such as tumor grade, stage, and completeness of surgical resection determine the administration of adjuvant chemotherapy in pediatric patients [7, 9–11]. The question of whether surgery only followed by a wait-and-monitor policy is sufficient, or if a multimodal treatment approach is necessary, has been an ongoing topic of discussion in previous studies conducted by the Children’s Oncology Group (COG), Children’s Cancer and Leukemia Group (CCLG), and Malignant Germ Cell International Collaborative (MaGIC) [10, 11, 14, 15]. The current study presents an Egyptian report on the management and outcomes of pediatric ovarian ITs, aiming to clarify our treatment strategy selection.

## Materials and methods

We conducted a retrospective review of the medical records of all pediatric patients with histologically confirmed pure ovarian ITs who were treated at Children’s Cancer Hospital – Egypt over a 15-year period (January 2008–January 2023). Patients over 18 years of age, those with yolk sac elements in ITs, other pathological types of ovarian tumors, or those lost during follow-up were excluded from the study. Children’s Cancer Hospital is the main institution for pediatric oncology services in our country, with a dedicated capacity of 360 beds for childhood tumors. The institutional review board of our hospital reviewed and approved the study design, with an approval code of 105/2023.

The collected data were analyzed to examine clinical characteristics, imaging studies, tumor staging and grading, operative details, pathological reports, adjuvant chemotherapy, events, and overall outcomes. At the time of presentation, serum tumor markers such as alpha feto-protein (AFP) and beta-human chorionic gonadotropin (B-HCG) were assessed. The COG ovarian staging system was used for tumor staging [16–18]. GP was defined as the implantation of mature glial tissue within the peritoneal cavity. In patients where peritoneal nodules of GP or mature teratomas were detected, tumor upstaging was not considered. However, the presence of peritoneal deposits of ITs resulted in upstaging.

Complete tumor resection (R0) was assigned when tumor resection with salpingo-oophorectomy or ovarian-sparing surgery was performed, ensuring that there were free resection margins. Microscopic positive margin (R1) was considered in the presence of microscopic residuals with capsular involvement. Peritoneal wash for cytological examination, along with a meticulous evaluation of the omentum, peritoneal surfaces, regional lymph nodes, and the contralateral ovary were essential for precise staging. The histological diagnosis of ITs was confirmed in the presence of immature neuroepithelial rosettes and tubules combined with mature tissue. The final histopathological results and grading were reported by our pediatric pathologists using the Norris histological grading system that is summarized in Table 1 [6]. Adjuvant chemotherapy was administered for patients with incompletely resected or locally advanced tumors. The combination of cisplatin, etoposide, and bleomycin (PEB) was the standard regimen for all patients. Patients were strictly monitored every three months during the first three years, and then every six months thereafter. Imaging scans and tumor markers were conducted at each follow-up visit. The follow-up data was collated until January 2024, and events were reported.

**Table 1:** The Norris histological grading system for immature teratoma [6]

Grade	Description
I	The amount of immature tissue present on any slide occupies up to one low-power microscopic field, but does not exceed the area of one low-power field
II	Immature tissue occupies more than one low-power microscopic field, but does not exceed three low-power microscopic fields
III	Immature tissue present on any slide extends beyond three low-power microscopic fields

We conducted the statistical analysis using SPSS, version 22 (Statistical Package for Social Science). The overall survival (OS) and event-free survival (EFS) were estimated using the Kaplan-Meier method. The probability of survival was provided with their standard errors (SE). OS was determined as the time interval from the date of diagnosis until the date of death. EFS was determined as the time interval from the date of diagnosis to the first event, including relapse or metachronous disease or death. The development of mature teratoma in any patient during the follow-up period was not considered an event.

## Results

### Clinical characteristics

**Fig. 1:**
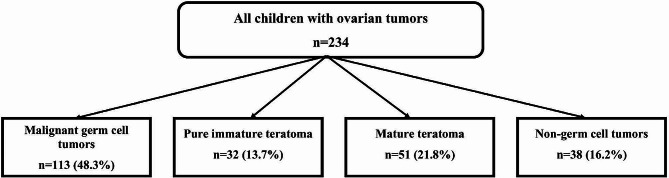
The different pathological types of ovarian tumors managed at our center throughout the study duration

**Fig. 2:**
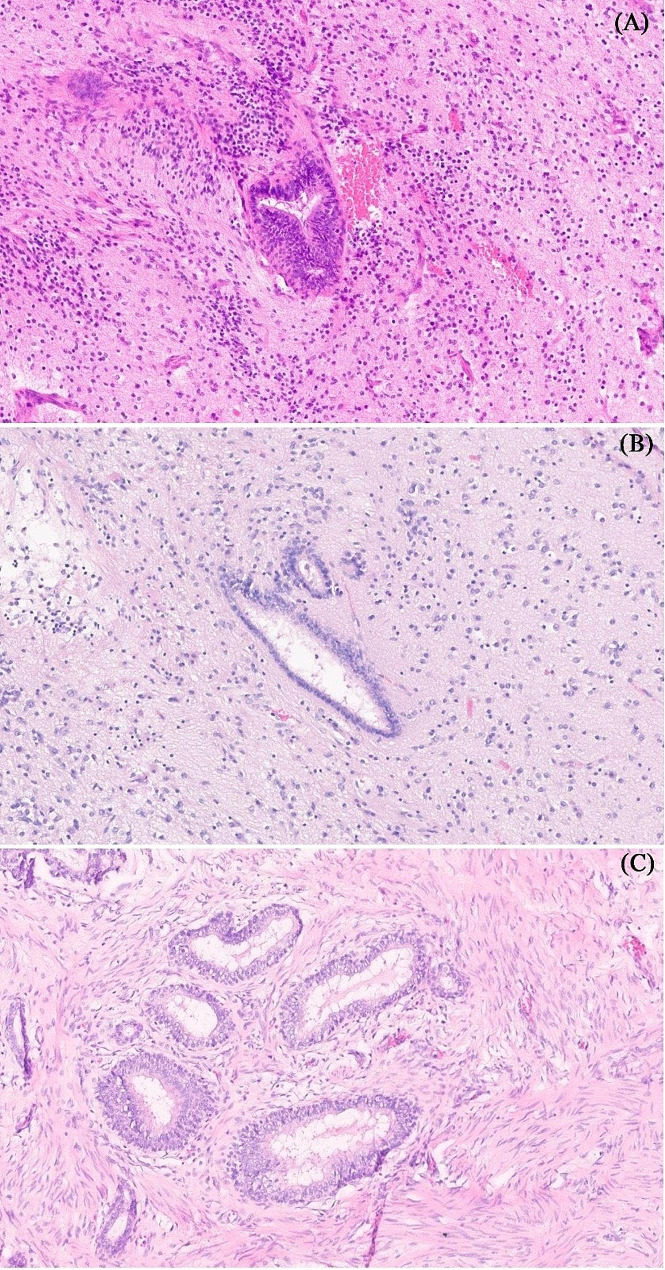
The histopathological distribution and description of the Norris grading system among the included patients; **(A**): Grade I, *n* = 16 (50%). Section shows glial tissue components of teratoma with one focus of neuroepithelial element representing Grade I IT (H&E stain, magnification x 400). **(B**): Grade II, *n* = 9 (28%). Section shows glial tissue components of teratoma with two foci of neuroepithelial elements representing Grade II IT (H&E stain, magnification x400). **(C)**: Grade III, *n* = 7 (22%). Section shows glial tissue components of teratoma with more than three foci of neuroepithelial elements representing Grade III IT (H&E stain, magnification x400)

During the study period, a total of 234 children with ovarian tumors were presented and treated at our institution. Among them, there were 32 patients (13.7%) who were diagnosed with pure ITs. Figure 1 demonstrates the different pathological types of ovarian tumors managed at our center throughout the study duration. The analysis focused on 32 children who had histologically confirmed pure ITs. The median age at presentation was 9 years, ranging from 10 months to 17 years. One patient presented as an emergency case with acute abdomen and ovarian torsion, whereas the remaining 31 patients were diagnosed at the elective oncology clinic. The most common presenting complaint was a palpable pelviabdominal mass with increased abdominal girth, observed in 20 patients (62.5%). Out of all patients, 97% had unilateral tumors, with a median tumor size of 9 cm in the largest dimension (range: 6–15 cm). All tumors were potentially resectable based on preoperative imaging evaluation. The assessment of serum tumor markers was documented in 27 patients. In 5 patients, the serum level of AFP was elevated, with a median of 215 ng/ml (range: 86–490). Regarding the final tumor grading, 16 patients (50%) were classified as grade I according to the Norris classification. Figure 2 shows the histopathological distribution and description of the Norris grading system among the included patients. The clinical characteristics of all patients with pure ITs are summarized in Table 2.

**Table 2:** The clinical characteristics of all patients with pure immature teratoma

Parameters	Number (%)
**Age group**	
Pre-pubertal	17 (53%)
Post-pubertal	15 (47%)
**Main presenting symptom**	
Palpable abdominal mass	20 (62.5%)
Chronic abdominal pain	11 (34.5%)
Acute abdomen and ovarian torsion	1 (3%)
**Tumor laterality**	
Unilateral	31 (97%)
Bilateral	1 (3%)
**Serum tumor Markers**	
Within normal range (AFP and HCG)	22 (68.8%)
Elevated (AFP only)	5 (15.7%)
Missed	4 (12.5%)
Not done	1 (3%)
**Staging according to COG**	
I	28 (87.5%)
II	1 (3%)
III	3 (9.5%)
IV	-
**Grading according to Norris classification**	
Grade I	16 (50%)
Grade II	9 (28%)
Grade III	7 (22%)

### Surgery and adjuvant therapy

Primary surgical resection, through an open approach, was performed for all included patients. Regarding the extent of resection, unilateral salpingo-oophorectomy was done in 31 patients. The patient diagnosed with bilateral tumors (measuring 11 cm and 6 cm respectively) underwent unilateral salpingo-oophorectomy on one side, while tumor enucleation (ovarian-sparing surgery) was performed on the smaller cystic lesion. Surgical staging procedures were completely carried out on all patients. Based on the COG staging, 28 patients (87.5%) were classified as stage I, and five of them had confirmed GP according to the post-operative pathological reports of the biopsies. None of the five patients with GP underwent extensive or additional surgical procedures. One patient was assigned as stage II due to the presence of microscopic residuals (R1) with capsular involvement after precise pathological assessment. Three patients were categorized as stage III; one of them had positive peritoneal cytology with IT peritoneal implants, which required a second-look surgery after 10 days for further resection. Intraoperative tumor rupture occurred in another patient, and the remaining patient had positive regional lymph nodes (more than 2 cm).

Twenty-eight patients (87.5%) were treated using surgery-alone strategy. The remaining four patients (12.5%) received adjuvant chemotherapy, and all of them were administered four cycles of PEB regimen. Among these patients, three were diagnosed as stage III (two had grade II tumors and one had a grade I tumor). The remaining patient was diagnosed as stage II and had a grade III tumor. During the course of adjuvant chemotherapy, one patient presented with clinical manifestations of adhesive intestinal obstruction which was managed by surgical exploration and adhesiolysis after failure of conservative treatment.

### Follow-up and outcomes

The follow-up time ranged between 15 and 188 months, with a median of 86 months. There were no deaths reported in the series, while two patients had tumor relapse/metachronous IT after finishing their treatment. The first patient, who was 12 years old at presentation, encountered intraoperative tumor rupture and classified as stage III/grade I. She completed post-operative chemotherapy, and continued under close monitoring. After 10 months, imaging studies revealed a mass on the operative bed that necessitated a second surgery to resect the tumor. After complete resection of the IT relapse with loops of the small bowel, she received a second-line chemotherapy (four cycles of vinblastine, bleomycin, and cisplatin). The patient is now in complete remission and has been followed up for 60 months, without any further events. The second patient, aged 10 years at time of diagnosis, was initially managed by surgery-alone approach for a stage I/grade I tumor. During follow-up imaging scans, a lesion on the contralateral ovary was discovered after 19 months of the primary surgery. Ovarian-sparing surgery was performed with negative resection margins and a final diagnosis of metachronous IT was reported. Thereafter, the patient received four cycles of the second-line chemotherapy. Currently, she is also in complete remission and has been followed up for 47 months. The 5-year OS for all included patients was 100% and the 5-year EFS was 93.4 ± 4.5%.

One patient, who was 7 years at presentation, had an initial stage I/grade I IT that was managed with surgery-alone approach and had a metachronous ovarian mass during follow-up. Ovarian-sparing surgery was carried out, and the final pathological assessment revealed a mature teratoma, which was not considered as an event. None of the five patients with GP that were detected at the primary surgery had tumor relapse or any oncological event until the end of the monitoring time.

## Discussion

The current study discusses the experience of an Egyptian pediatric oncology center in managing pure ovarian ITs, which represented 13.7% of all ovarian tumors treated over 15 years. Previous studies reported incidences of pure ovarian ITs ranging from 3 to 11.1% among all ovarian neoplasms in this age group [1, 19, 20]. However, Shinkai and colleagues observed a higher incidence of 26.9% for pure ovarian ITs in their study [5]. In our study, grade I ITs were the most common among all patients, accounting for 50% of the cases. A similar finding was reported by Pavone et al., who reported that grade I ITs were also the most prevalent, representing 44% of all their patients [9]. On the contrary, two previous studies reported that grade III ITs were the most common among their cases, constituting 42.9% and 39%, respectively [5, 11]. These disparities in the findings regarding the most common grade of ITs could be attributed to differences in referral patterns and the varying numbers of patients included in each study. We believe that the incidences of ovarian ITs and the prevalence of different grades are not yet precisely known due to the paucity of such tumors in pediatric age. Further research with a large sample size would be required to provide more accurate data.

There is a difference in the classification of ovarian ITs between adults and children [5]. These neoplasms are typically considered as malignant germ cell tumors in adult women [12, 13]. However, in girls, there is current controversy concerning the malignancy potential of ITs, and grade I tumors are considered non-malignant [21, 22]. As a consequence to this uncertainty, the treatment strategy in children differs from that in adults [7, 9–13]. The administration of post-operative chemotherapy in ITs is still questionable, and this highlights the difference in treatment approaches between adult and pediatric oncologists [9]. In this study, we applied a surgery-alone approach followed by close observation to all patients with tumors confined to the ovary, regardless of the grade. This strategy achieved excellent outcomes among our patients. The same treatment strategy has been successfully implemented in previous studies conducted on pediatric cohorts [5, 15, 21, 23]. This strategy is based on the observation that ovarian ITs are not chemosensitive in children [24], and adjuvant chemotherapy had no significant effect in reducing the incidences of relapse among pediatric patients [15, 21, 25]. Another argument for this strategy is that the risk of tumor relapse varies depending on the staging, with a higher risk observed in stages III and IV compared to stages I and II [10, 25]. In this study, adjuvant chemotherapy was exclusively administered for patients with stages II and III. We believe that incomplete tumor removal and extension of the tumor outside the ovary are the most important risk factors for relapse, as previously reported [5, 26].

Recently, a Brazilian study by Vieira et al. included 42 children with ovarian ITs (29 treated by surgery alone and 13 received adjuvant chemotherapy). They reported no significant differences in the relapse rates and overall outcomes between patients who received adjuvant chemotherapy and those who did not. Furthermore, Vieira et al. compared the Brazilian cohort to 98 children in the INT-0106/GC-2 trials from the United States and United Kingdom. They observed no significant difference in the survival outcomes, despite 87% of stage II–IV Brazilian patients received adjuvant chemotherapy compared to only 13% of patients in the United States and United Kingdom cohorts [27]. According to some previous reports, complete surgical resection of the gross tumor followed by observation is sufficient in pediatric patients, irrespective of both grading and staging [14, 28]. A recent review on the management of pediatric and adult patients with ovarian ITs recommended strict follow-up alone after surgical resection for these children at the initial presentation and even if tumor relapse does occur [29]. Pashankar and colleagues documented in their study that tumor grading is the most significant factor for relapse. They found that children with grade I ITs had no relapses, whatever tumor staging or age. Moreover, children with grade III tumors had the highest incidence of relapse, with a rate of 21% [11]. However, contrary findings were observed in our study, as no relapses were reported among children with grade III tumors. The occurrence of tumor rupture, either preoperative or during surgery, is a challenge in achieving optimal outcome. In our study, one patient experienced intraoperative tumor rupture, and received post-operative chemotherapy. However, tumor relapse occurred in spite of completing the course of adjuvant chemotherapy. In contrast, Pavone et al. reported in their study that they had 13 patients with ruptured ITs, and only 5 of them received adjuvant chemotherapy. However, no events happened among these 13 patients, whether they received or did not receive post-operative chemotherapy. They concluded that adjuvant chemotherapy is not necessary for ruptured tumors [9]. These discrepancies between our series and the aforementioned studies highlight the need for additional investigations to identify the relationship between tumor grading, staging and relapse rate. Furthermore, the potential role of adjuvant chemotherapy in patients with ruptured ITs requires to be meticulously addressed.

Peritoneal spread in children with ovarian ITs presents with deposits of either IT or mature teratoma or GP, which is considered reactive rather than malignant [7, 8]. Some reports indicated that patients with GP at diagnosis often develop frequent relapses, requiring extensive surgical procedures to obtain final favorable results [15, 30]. In contradiction to these studies, we had five patients who presented with GP, and none of them underwent additional surgery beyond biopsy. These 5 patients did not receive adjuvant chemotherapy and did not show any signs of progressive disease or oncological events. On the other side, we had one patient who was presented with IT deposits that required extensive surgery for resection. Our findings were in line with other studies which suggested that a conservative surgical approach is sufficient for ITs with GP, whereas extensive resection is crucial for IT deposits to achieve long-term disease-free survival [9, 31].Thus, we believe that an accurate histological examination of peritoneal deposits is mandatory for guiding the appropriate surgical strategy. Eventually, our study has limitations such as being a retrospective single-center report that analyzes a relatively limited number of patients.

## Conclusions

Surgery-alone strategy with close follow-up achieves excellent outcomes for localized ovarian ITs in children, irrespective of the Norris grading or the presence of GP. However, adjuvant chemotherapy is questionable for patients with incompletely resected or locally advanced tumors, and its role requires further evaluation through prospective multicentric studies with a larger sample size.

## Data Availability

No datasets were generated or analysed during the current study.
